# Account of Diseases in an Eastern District, from the 20th of September to the 20th of October

**Published:** 1799-11

**Authors:** 


*Account of Diseases in an Eastern District, from the 20th of September to the 20th of October.*

| | No. of Cases. | | No. of Cases. |
|---|---|---|---|
| ACUTE DISEASES. | | Dysenteria - - | 6 |
| Typhus Mitior - - | 5 | Cholera Morbus - | 4 |
| Peripneumonia - - | 2 | Hæmorrhois - - | 2 |
| Hæmoptysis - - - | 2 | Colica Pictonum - - | 3 |
| Quotidian - - - | 2 | Menorrhagia - - | 4 |
| Acute Rheumatism - - | 4 | Amenorrhœa - - | 5 |
| CHRONIC DISEASES. | | Chlorosis - - - | 7 |
| Cough - - | 14 | Dysuria - - - | 5 |
| Dyspnœa - - | 8 | Hysteria - - - | 6 |
| Cough and Dyspnœa - | 12 | Hypochondriasis - | 5 |
| Phthisis Pulmonalis - | 6 | Psora - - - | 1 |
| Hydrothorax - - | 2 | Prurigo - - | 3 |
| Pleurodynia - - | 3 | PUERPERAL DISEASES. | |
| Palpitatio - - | 2 | Low Fever - - | 3 |
| Leipothymia - - | 1 | Ephemera - - - | 4 |
| Cephalœa - - - | 5 | Mastodynia - - | 7 |
| Vertigo - - | 3 | Menorrhagia lochialis - | 4 |
| Epilepsia - - | 2 | INFANTILE DISEASES. | |
| Apoplexia - - - | 1 | Hooping Cough - - | 5 |
| Paralysis - - | 2 | Measles - - - | 8 |
| Dyspepsia - - | 7 | Ophthalmia - - | 3 |
| Gastrodynia - - | 5 | Ophthalmia purulenta - | 2 |
| Diarrhœa - - | 13 | | |

